# Effects of fermented bamboo fiber on intestinal health and fecal pollutants in weaned piglets

**DOI:** 10.3389/fnut.2025.1538560

**Published:** 2025-03-31

**Authors:** Yubiao Jia, Qiuming Huang, Rui Song, Yanling Tang, Mengxin Feng, Jianjun Lu

**Affiliations:** ^1^Feed Science Institute, College of Animal Science, Zhejiang University, Hangzhou, China; ^2^Key Laboratory of Animal Nutrition and Feed Science in East China, College of Animal Science, Zhejiang University, Hangzhou, China

**Keywords:** weaned piglet, intestinal health, fecal pollutant, weaning stress, fermented bamboo fiber

## Abstract

**Introduction:**

Weaning stress adversely affects piglet growth and development, thereby reducing the economic efficiency of pig farming operations. Furthermore, pig feces are a major source of environmental pollution, underscoring the need for effective strategies to mitigate fecal output at its source.

**Methods:**

This study investigated the effects of dietary supplementation with fermented bamboo fiber (FBF) on growth performance, intestinal barrier integrity, gut microbiota composition, and fecal pollutant levels in weaned piglets. A total of 144 Duroc × Landrace × Yorkshire piglets, weaned at 21 days of age, were randomly assigned to 4 groups, with six replicates per group and 6 piglets per replicate. The control group (CON) received a basal diet, while the three treatment groups were fed the basal diet supplemented with 1, 1.5, and 2% FBF, respectively. The trial lasted 30 days.

**Results:**

The findings revealed that FBF supplementation fortified the intestinal barrier, modulated colonic microbial communities, and decreased fecal pollutant levels. Among the treatment groups, supplementation with 1.5% FBF produced the most significant improvements in piglets’ growth performance and intestinal barrier function, as well as the strongest microbial interactions and the greatest reduction in fecal pollutants.

**Discussion:**

These results suggest that FBF supplementation can alleviate weaning stress and mitigate the environmental impact of pig feces, with 1.5% identified as the optimal supplementation level.

## 1 Introduction

With the ongoing modernization of agriculture and the growth of large-scale farming, early weaning at 21 days has become a common practice in modern pig production. In intensive farming systems, piglets are typically weaned at significantly earlier ages (ranging from 3 to 5 weeks) compared to the natural weaning age of approximately 17 weeks (1). This early weaning period represents a critical phase in swine production, during which the animals are exposed to a range of stressors. Piglets undergo significant social and environmental changes, including separation from their mothers and littermates (2). Moreover, they must rapidly adjust to abrupt alterations in both their diet and housing conditions (3), often resulting in transient periods of hypo- or anorexia (4). This occurs during a stage when piglets’ immune systems are still immature (5), thermoregulation is limited (6), and digestive function is underdeveloped (7), in conjunction with an unstable intestinal microbiota (8). Consequently, weaning represents a period of considerable challenge for the animals, significantly impacting their performance (9).This complex sequence of physiological and environmental stressors is widely recognized as post-weaning syndrome, a condition that has been extensively studied and reviewed (2, 7, 10). The traditional approach to overcome this situation has been the use of in-feed antibiotics. However, in China, the use of antibiotics as growth promoters has been banned, and worldwide authorities are also pressing to limit its therapeutic use. In light of these challenges, both the swine industry and researchers have been actively exploring a range of strategies, including biosecurity measures (11), management practices (10, 12), genetic improvements (13), and nutritional interventions (9, 14), aimed at mitigating the adverse effects of weaning on piglets. Furthermore, the management of livestock manure has emerged as a significant environmental concern, as improper handling can pose substantial challenges to the sustainable development of the livestock industry. The inefficient use of nitrogen, phosphorus, and sulfur in pig diets results in these elements being primarily excreted in compound forms through feces, further exacerbating environmental pollution (15, 16). The odorous compounds emitted from feces not only diminish livestock production performance but also pose health risks such as respiratory distress and illnesses for farm workers and nearby residents (17–19). High levels of these odorous emissions can even lead to serious ecological disturbances, contributing to issues such as acid rain and nitrification (20, 21). Therefore, reducing pollution from livestock at its source is an important strategy for promoting ecological farming.

China is the leading global producer of bamboo, encompassing approximately 44 genera and 300 species, which together cover roughly 3% of the world’s forested area. Notably, Moso bamboo (*Phyllostachys edulis*) accounts for around 74% of the total bamboo forested area (22, 23). Moso bamboo is characterized by its rapid growth, short maturation period, and robust natural regeneration capacity (24, 25). It is abundantly available, readily accessible, and economically viable, rendering it a promising fiber source for animal feed (26, 27). Furthermore, dietary fiber has been shown to mitigate the emission of fecal pollutants. Research indicates that a high-fiber diet is associated with reduced urinary excretion of phosphorus, calcium, sodium, and sulfate (28). In contrast, inadequate dietary fiber intake leads to diminished colonic short-chain fatty acid (SCFA) production, with protein fermentation potentially generating harmful metabolites, including skatole, indole, and phenols. The release of these substances may exceed the tolerance thresholds of both humans and animals, thereby disrupting normal physiological and production processes (15). Fermented bamboo fiber (FBF) is produced through the microbial fermentation of bamboo fiber using specific bacteria (Lactic acid bacteria, yeast, and *Bacillus subtilis*). As a heterogeneous carbohydrate, bamboo fiber resists enzymatic digestion in the mammalian small intestine and is primarily fermented by microbes in the colon (29). Numerous studies indicate that inadequate dietary fiber intake is correlated with dysbiosis of the gut microbiota, which can contribute to conditions such as intestinal inflammation, colon cancer, obesity, and type II diabetes (30–32). Consequently, the supplementation of dietary fiber has been extensively studied for its potential to restore microbial balance, promote gut health, and modulate immune responses (33, 34). Furthermore, microbial fermentation of feed has been shown to decrease antinutritional factors, such as *β*-conglycinin and glycinin (35), while improving the nutritional profile by increasing levels of crude protein, total protein, and unsaturated fatty acids (36). Huangfu et al. (37) also found dietary fiber could alleviate weaning stress in piglets by reducing intestinal inflammation and repairing intestinal barrier function. This study, therefore, aims to evaluate the effects of FBF on the growth performance and adverse reactions in post-weaning piglets, which can contribute to a better understanding of its role in promoting health at this critical developmental stage.

In our previous research, we assessed the effects of FBF on gestating sows and nursing piglets (26). The findings indicated that incorporating this fiber into their diet reduced backfat loss in lactating sows, improved gut health, and enhanced weight gain in piglets. These results suggest that FBF plays a beneficial role in promoting swine growth performance and gastrointestinal health. However, a key question remains as to how FBF supplementation might affect the growth performance, intestinal barrier function, and gut microbiota of weaned piglets, which has yet to be systematically evaluated. Additionally, we previously observed that adding FBF to the diet could mitigate odor levels in pig housing, suggesting a potential reduction in pollutants present in pig manure. While promising, this observation has not been subjected to rigorous empirical testing, and there is only limited research on the relationship between dietary fiber and manure pollutants, resulting in an incomplete understanding of its environmental effects. Consequently, this study aims to elucidate the effects of FBF on growth performance, gut health, and fecal pollutants in piglets, while determining its optimal supplementation levels. If verified, the benefits and recommended levels of FBF could provide a robust theoretical foundation for its application, leading to various downstream benefits, such as alleviating weaning stress, enhancing growth performance, improving gut health, and mitigating environmental pollution in livestock farming.

## 2 Materials and methods

### 2.1 Fermented bamboo fiber

Moso bamboo (Phyllostachys) used in this experiment with an age of 2 to 3 years, was obtained from Zhejiang Province, China. The general bamboo powder production process involved chopping, crushing, and sieving with 40 mesh. The fermentation substrate consisted of bamboo fiber powder and 1% glucose, which was supplemented with 3 probiotics. The components of FBF are listed in Table 1.

**Table 1:** Nutritional composition of fermented bamboo fiber (FBF).

Items	Fermented bamboo fiber
DM (%)	88.32
CP (%)	1.73
CEE (%)	0.38
ASH (%)	1.24
NDF (%)	81.38
ADF (%)	61.48
CF (%)	81.04
Lignin (%)	15.46
Ca (%)	0.44
TP (%)	0.31
Water hold capacity, g/g	6.40
Water absorption and swelling properties, ml/g	7.50

### 2.2 Experimental design and diets

A total of 144 female weaned piglets (Duroc × Landrace × Yorkshire), aged 21 days and weighing 6.50 ± 0.61 kg, were randomly divided into four groups, with six replicates per group and six piglets per replicate. The experimental weaned piglets were sourced from a commercial pig farm in Jinhua, Zhejiang Province, China. The control group was received a basal diet, while the experimental groups were fed the basal diet supplemented with 1.0% (FBF-1 group), 1.5% (FBF-2 group), and 2.0% (FBF-3 group) fermented bamboo fiber. The experimental diets were formulated to maintain nutritional equivalence (excluding crude fiber) with the basal diet. All piglets were housed in a single facility under controlled environmental conditions, with temperatures maintained at 26–28°C and relative humidity between 40 and 60%. Following a 7-day pre-feeding period, a 30-day formal experimental period commenced, during which pigs had ad libitum access to feed and water. The basal diet, based on corn-soybean meal, was formulated according to the nutritional requirements for weaned piglets as per the National Research Council (NRC) 2012 standards. Detailed diet formulation and nutritional levels are presented in Table 2. All pigs were provided the diet and water ad libitum during the 30-day experiment.

**Table 2:** The dietary formulation and nutritional level were tested (DM basis).

Ingredients	Content			
	Basic diets	1.0% (FBF-1)	1.5% (FBF-2)	2.0% (FBF-3)
Corn	62.64	61.14	59.96	58.95
Soybean meal	18.00	18.00	18.00	18.00
Extruded soybean	10.00	10.00	10.50	10.80
Soybean oil	1.50	2.00	2.20	2.40
Fish meal	4.00	4.00	4.00	4.00
FBF	0	1.00	1.50	2.00
NaCl	0.25	0.25	0.25	0.25
CaHPO_4_	1.20	1.20	1.20	1.20
Limestone	1.00	1.00	1.00	1.00
Lysine • HCl	0.30	0.30	0.29	0.29
Met	0.03	0.03	0.03	0.04
Thr	0.08	0.08	0.07	0.07
Premix^1^	1.00	1.0	1.0	1.0
Total	100.00	100.00	100.00	100.00
Nutrient levels^2^				
DE/(MJ/kg)	13.95	13.94	13.95	13.95
CP (%)	19.18	19.15	19.20	19.18
Ca (%)	0.89	0.89	0.88	0.87
CF (%)	2.61	3.38	3.84	4.18
TP (%)	0.65	0.66	0.65	0.67
AP (%)	0.43	0.44	0.43	0.45
Lys (%)	1.23	1.22	1.22	1.23
Met + Cys (%)	0.72	0.73	0.72	0.72
Thr (%)	0.86	0.86	0.85	0.85

^1^The mineral premix provided the following per kg of diet: VA 8000 IU, VB1 4 mg, VB2 3.6 mg, VB5 40 mg, VB6 4 mg, VB12 0.02 mg, VD3 3,000 IU, VE 20 IU, VK3 2 mg, Biotin 0.15 mg, Folic acid 1.0 mg, D-pantothenic acid 11 mg, Nicotinic acid 10 mg, Antioxidant 100 mg, Cu (as copper sulfate) 10 mg, Fe (as ferrous sulfate) 80 mg, Mn (as manganese sulfate) 30 mg, Zn (as zinc sulfate) 75 mg, I (as potassium iodide) 0.40 mg, Se (as sodium selenite) 0.30 mg. ^2^DE and AP were calculated values and others were measured values.

### 2.3 Sample collection

Initial weights of the weaned piglets were recorded prior to the commencement of the study. Throughout the experimental phase, feed intake was regularly measured for each experimental unit. At conclusion of the trial, final weights were documented to calculate average daily gain (ADG), average daily feed intake (ADFI), and feed conversion ratio (F/G = ADFI/ADG). On the 29th day of the experiment, one piglet from each replicate (six piglets per replicate) was randomly selected for the collection of fresh feces to determine the levels of total nitrogen (TN), total phosphorus (TP), total sulfur (TS), indole, and skatole. At the end of the 30-day experiment, one piglet from each replicate (six piglets per replicate) was selected for slaughter. Serum samples were collected, aliquoted, and stored at −80°C for subsequent analysis. Samples of the duodenum, jejunum, ileum, colon segments, mucosa, and their contents were collected, flash-frozen in liquid nitrogen, and stored at −80°C for further testing.

### 2.4 Intestinal mucosal permeability

The levels of D-lactic acid (D-LA), diamine oxidase (DAO), intestinal trefoil factor (ITF), intestinal fatty acid binding protein (iFABP), endotoxin (ET) in serum were determined by a SpectraMax M5 microplate reader (Molecular Devices, Sunnyvale, CA, United States) using commercial ELISA kits (Shanghai Enzyme-linked Biotechnology Co., Ltd., Shanghai, China) according to the manufacturer’s protocol.

### 2.5 Intestinal morphology under microscope

Specimens of the duodenum, jejunum, and ileum were fixed in 4% neutral buffered formalin and processed using conventional histological techniques, including paraffin embedding. Paraffin blocks were sectioned into 6 μm slices and stained with hematoxylin and eosin (H&E). All samples were examined under a light microscope (Nikon Eclipse E-400) equipped with a digital camera head (DS-5 M) and camera control unit (DS-L1) from Nikon. Villi height and crypt depth were measured using image analysis systems. For transmission electron microscopy (TEM), specimens of the duodenum, jejunum, ileum, and colon were initially fixed with 2.5% glutaraldehyde (BL910A, Biosharp), followed by 1% osmium tetroxide (OsO4) fixation for 1–2 h. Subsequent steps included dehydration, infiltration, embedding, ultrathin sectioning, and staining. Images were captured using a TEM (Hitachi, Model H-7650).

### 2.6 DNA extraction and 16S rRNA gene sequencing

Genomic DNA from the sample was extracted using the CTAB method, and the purity and concentration of the DNA were tested. The DNA quality was determined by agarose (BIOWAST) gel electrophoresis, DNA concentration range: 47.2–96.5 ng/μl, DNA purity (A260/A280): 1.83–1.96. The microbial 16S rRNA gene was amplified targeting the V3-V4 variable regions, the primers were 341F(5’-CCTAYGGGRBGCASCAG-3′) and 806R(5’-GGACTACNNGGGTATCTAAT-3′). After amplification, the PCR products were purified following strict protocols as per the AXYGEN company kit instructions. Next Fluorescence quantification of PCR products was conducted, and products were mixed in appropriate proportions based on electrophoresis results. Next step is MiSeq Library Preparation and Sequencing Analysis, libraries were prepared using the NEBNext Ultra DNA Library Prep Kit and quality control was performed using Agilent Bioanalyzer 2,100 and Qubit. Sequencing was conducted on the MiSeq platform, and paired-end (PE) reads were assembled based on overlap relationships. Sequences underwent quality control and filtering, followed by operational taxonomic units (OTUs) analysis. Diversity indices, taxonomic classification, and OTU clustering analysis were performed. Community structures at various taxonomic levels were analyzed, and statistical tests for significant differences and multivariate analyses were conducted to assess community composition and phylogenetic information across multiple samples. 16S rRNA sequencing was conducted by Shanghai Majorbio Bio-pharm Technology Co., Ltd.^1^

### 2.7 Measurement of serum indicators about fecal constituents

The levels of parathyroid hormone (PTH), 1,25-dihydroxyvitamin D_3_ (1,25(OH)_2_D_3_), serum skatole, and indoleacetic acid decarboxylase (IAD) were measured using ELISA kits from Shanghai Enzyme-linked Biotechnology Co., Ltd. Serum inorganic phosphorus (IP) content was determined using the serum inorganic phosphorus assay kit from Beibo Biological. Procedures followed the instructions provided.

### 2.8 Analysis of fecal constituents

Fecal pH was measured using a benchtop pH meter (Lu Heng Biotechnology Co., Ltd., model: LH-P800). Fecal urease activity was measured using the solid-urease assay kit (Catalog number: A121-1-1 Solid-Urease, S-UE) from Nanjing Jiancheng Bioengineering Institute. Total nitrogen (TN) in feces was determined using the Kjeldahl method. Total phosphorus (TP), organic phosphorus (OP), and inorganic phosphorus (IP) in feces were measured using the molybdenum blue colorimetric method with the soil total phosphorus/organic phosphorus/inorganic phosphorus assay kit (Catalog number: AKEN036C) from Beijing Hezi Bio-Technology Co., Ltd. Total sulfur was determined using the turbidimetric method by Wuhan Purnas Biotechnology Co., Ltd. Fecal indole and skatole content were measured by Wuhan Punes Biotechnology Co., Ltd.

### 2.9 Data statistics and analysis

All data in the current study were initially processed and analyzed using Excel 2016 for basic statistical calculations. Multiple group comparisons were conducted using SPSS Statistics 22.0 (SPSS Inc., Chicago, IL) with one-way analysis of variance (ANOVA), followed by Duncan’s *post hoc* test. Comparisons between two groups were analyzed using Student’s t-test. Experimental data are presented as Mean ± SEM, and statistical significance was considered at *p* < 0.05. Correlation analysis was performed using the Spearman correlation test. Graphs were generated using GraphPad Prism 9.

## 3 Results

### 3.1 Effects of supplementation with fermented bamboo fiber on the growth performance of piglets

**Figure 1:**
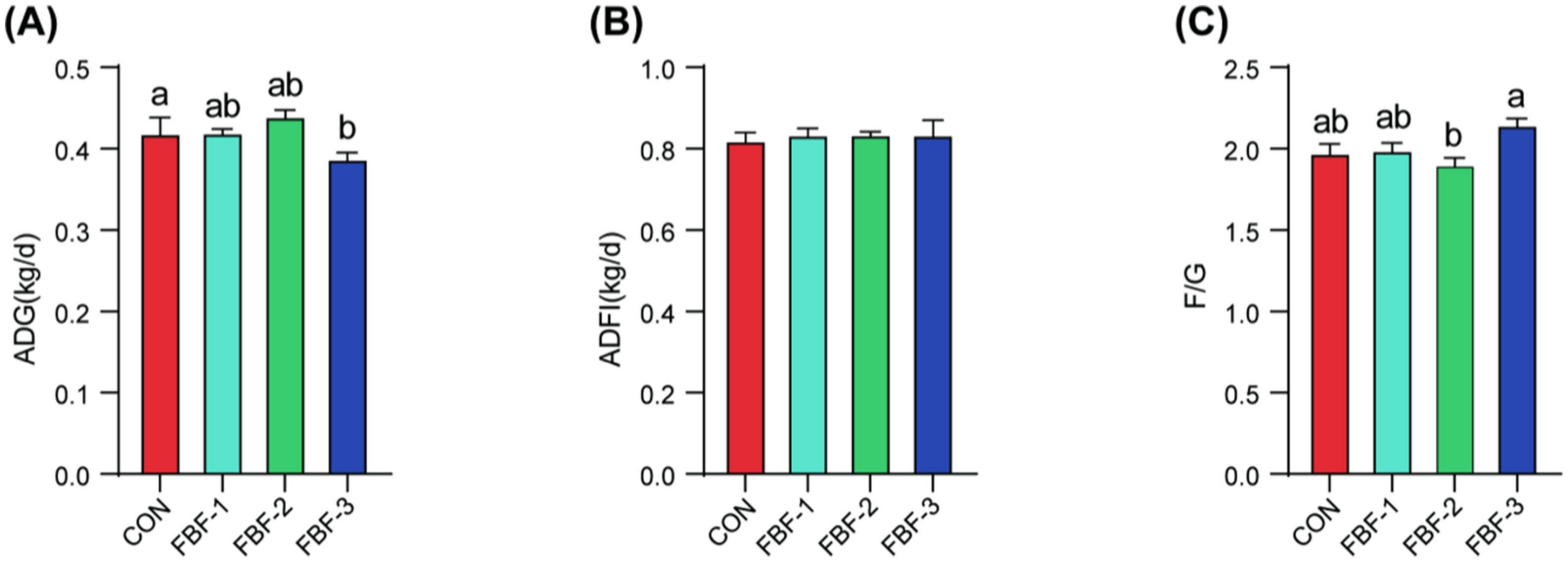
The effects of FBF supplementation on the growth performance of weaned piglets. **(A)** ADG (Average daily gain), (B) ADFI (Average daily feed intake), (C) F/G (Feed to gain ratio). Different letters indicate significant differences (*p* < 0.05).CON Basic diets; FBF-1, 1% fermented bamboo fiber diet; FBF-2, 1.5% fermented bamboo fiber diet; FBF-3, 2% fermented bamboo fiber diet.

The impact of dietary supplementation of fermented bamboo fiber on growth performance, with a notable difference at the 1.5% concentration, is depicted in Figure 1, highlighting the optimal level of FBF supplementation. Over the 30-day study period, piglets in the FBF-2 group exhibited the highest average daily gain, although this difference did not reach statistical significance. This group also demonstrated the lowest feed conversion ratio, which was significantly lower than that observed in the FBF-3 group (*p* < 0.05). In contrast, the FBF-3 group showed a significant reduction in average daily gain (*p* < 0.05) and an increase in the feed conversion ratio compared to the control group. No significant differences were noted in average daily feed intake across all groups.

### 3.2 Effects of supplementation with fermented bamboo fiber on the intestinal morphology

**Figure 2:**
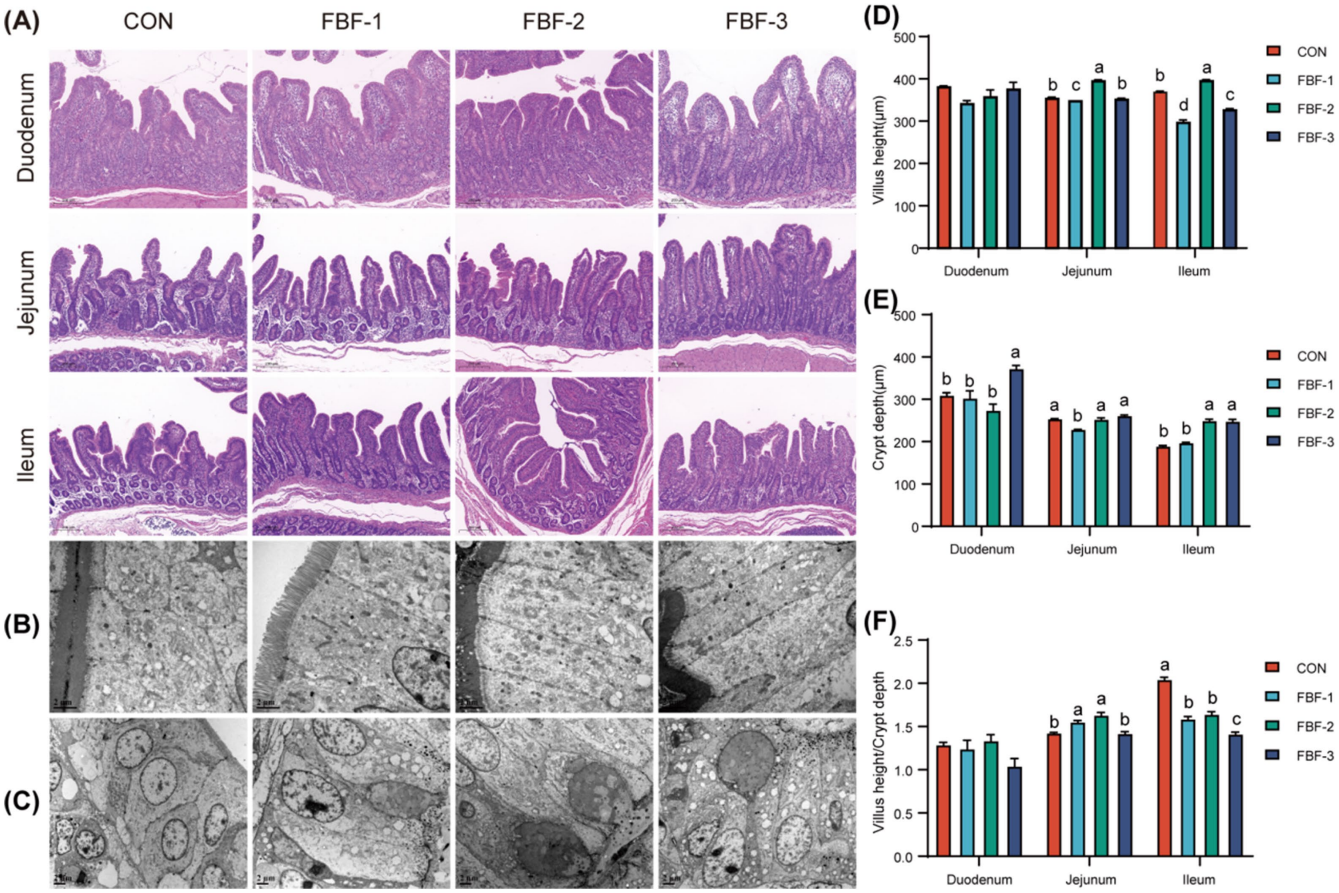
Hematoxylin and eosin (H&E) staining results of the small intestine of weaned piglets, scale bars: 200 μm **(A)**. The ultrastructural observation of jejunum and colon by transmission electron microscope **(B,C)**. The structure of the jejunum was observed by transmission electron microscopy (TEM), scale bars: 10,000×, 2 μm **(B)**. The structure of colon was observed by TEM, scale bars: 5,000×, 2 μm **(C)**. Effect of FBF supplementation on the intestinal morphology of weaned piglets **(D–F)**. **(D)** Villus height (VH); **(E)** Crypt depth (CD); **(F)** Villus height/crypt depth (V/C). Different letters indicate significant differences (*p* < 0.05).

Hematoxylin and eosin (H&E) staining of the piglet small intestine (Figure 2A) demonstrated that the villi in all experimental groups exhibited a more compact and uniform architectural arrangement compared to the control group. Morphometric analysis of the intestinal villi (Figures 2D–F) demonstrated that the FBF-2 group significantly increased villus height in the jejunum and ileum (*p* < 0.05), reduced crypt depth, and enhanced the villus-to-crypt ratio in the jejunum relative to the control. Conversely, the FBF-3 group exhibited reduced villus height and villus-to-crypt ratio in the small intestine while showing an increase in crypt depth. Transmission electron microscopy (TEM) observations of the jejunal and colonic epithelial cells (Figures 2B,C) indicated that the experimental groups had more distinctly defined epithelial boundaries and more orderly cellular arrangements compared to the control, with the FBF-2 group displaying the most compact epithelial structure (Figure 2B). Colonic cells in the experimental groups were also more compact, with more intact nuclei and clearer secretory glands (Figure 2C).

### 3.3 Effects of supplementation with fermented bamboo fiber on intestinal barrier

**Figure 3:**
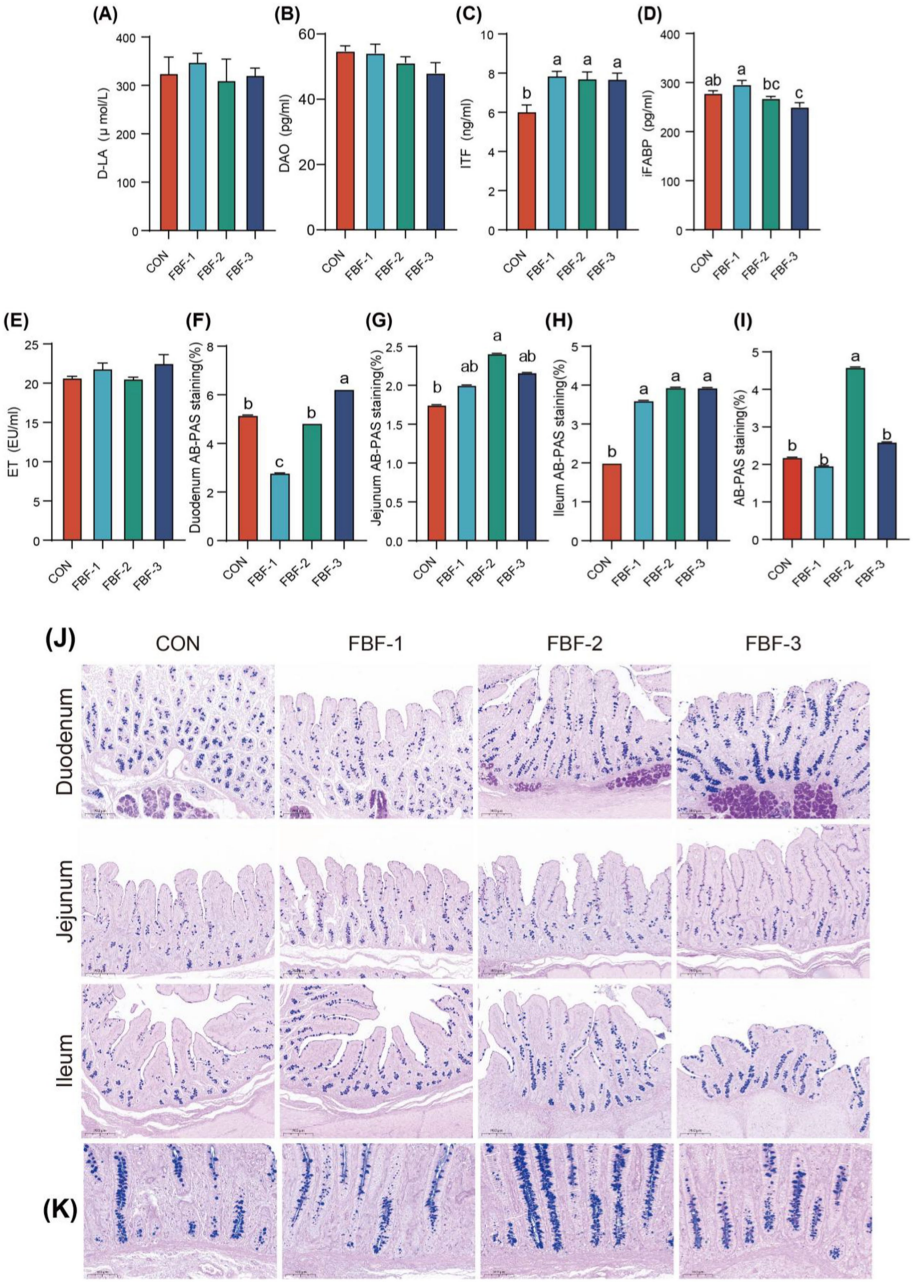
Effects of FBF supplementation on serum intestinal barrier indicators **(A–E)** in weaned piglets, D-lactic acid (D-LA), diamine oxidase (DAO), intestinal trefoil factor (ITF), intestinal fatty acid binding protein (iFABP), endotoxin (ET). Duodenal, jejunum, ileum Alcian blue-periodic Acid Schiff (AB-PAS) staining, blue is acidic mucus, positive staining, scale bars = 200 μm **(J)**. Percentage of the positive relative staining area of the duodenum **(F)**, jejunum **(G)**, ileum **(H)**. Effects of FBF supplementation on colon acidic mucus secretion of weaned piglets **(K)**, Scale bars: 20×, 100 μm, Percentage of the positive relative staining area of the colon **(I)**.

The effects of dietary fermented bamboo fiber on serum intestinal barrier indicators in piglets are presented in Figures 3A–E. Supplementation with varying concentrations of fermented bamboo fiber resulted in a significant increase in serum ITF levels. Notably, serum iFABP concentrations were highest in the FBF-1 group, significantly exceeding those observed in the FBF-2 and FBF-3 groups (*p* < 0.05). In contrast, serum levels of D-LA, DAO, and ET remained unaffected by the fiber supplementation. Figure 3J illustrates the effect of fermented bamboo fiber on mucin secretion by goblet cells in the piglet small intestine, where mucin distribution was markedly greater in the experimental groups compared to the control group, increasing in proportion to the amount of fiber added. Further statistical analysis (Figures 3F–H) revealed that mucin content in the jejunum was higher in all experimental groups than in the control group, with the FBF-2 group showing a significant increase. Similarly, mucin content in the ileum was significantly elevated in the experimental groups relative to the control group. Mucin distribution and content in the colon are depicted in Figures 3I,K, indicating a significantly higher mucin content in the FBF-2 group compared to the other groups.

### 3.4 Effects of supplementation with fermented bamboo fiber on colonic microbiota of piglets

**Figure 4:**
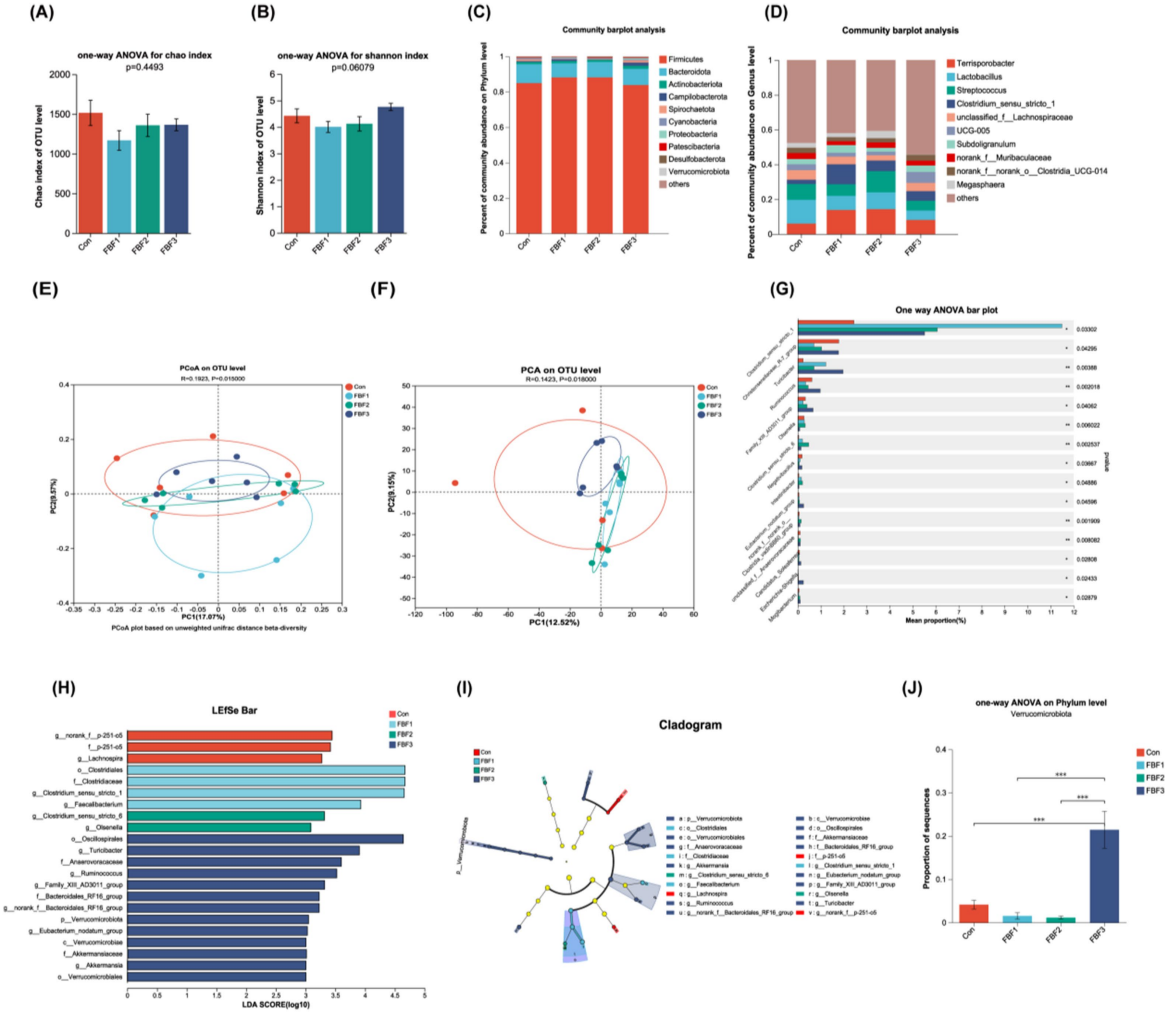
Analysis of colon microbial *α*-diversity and colon microbial *β*-diversity in weaned piglets. **(A)** Chao index of OUT level, **(B)** Shannon index of OUT level, **(E)** Principal coordinate analysis based on Unweighted Unifrac distance, **(F)** Principal component analysis. Analysis of colon microbial composition and LEfSe analysis of colon microorganisms in weaned piglets. **(C)** Microbial composition at the phylum level, **(D)** Microbial composition at the genus level, **(J)** Differential bacteria proportion of sequence at the phylum level, **(G)** Differential bacteria proportion of sequence at the genus level, **(I)** LEfSe cladogram, **(H)** Linear discriminant analysis.* indicates statistical differences between groups when *p < 0.05, **p < 0.01, ***p < 0.001.

**Figure 5:**
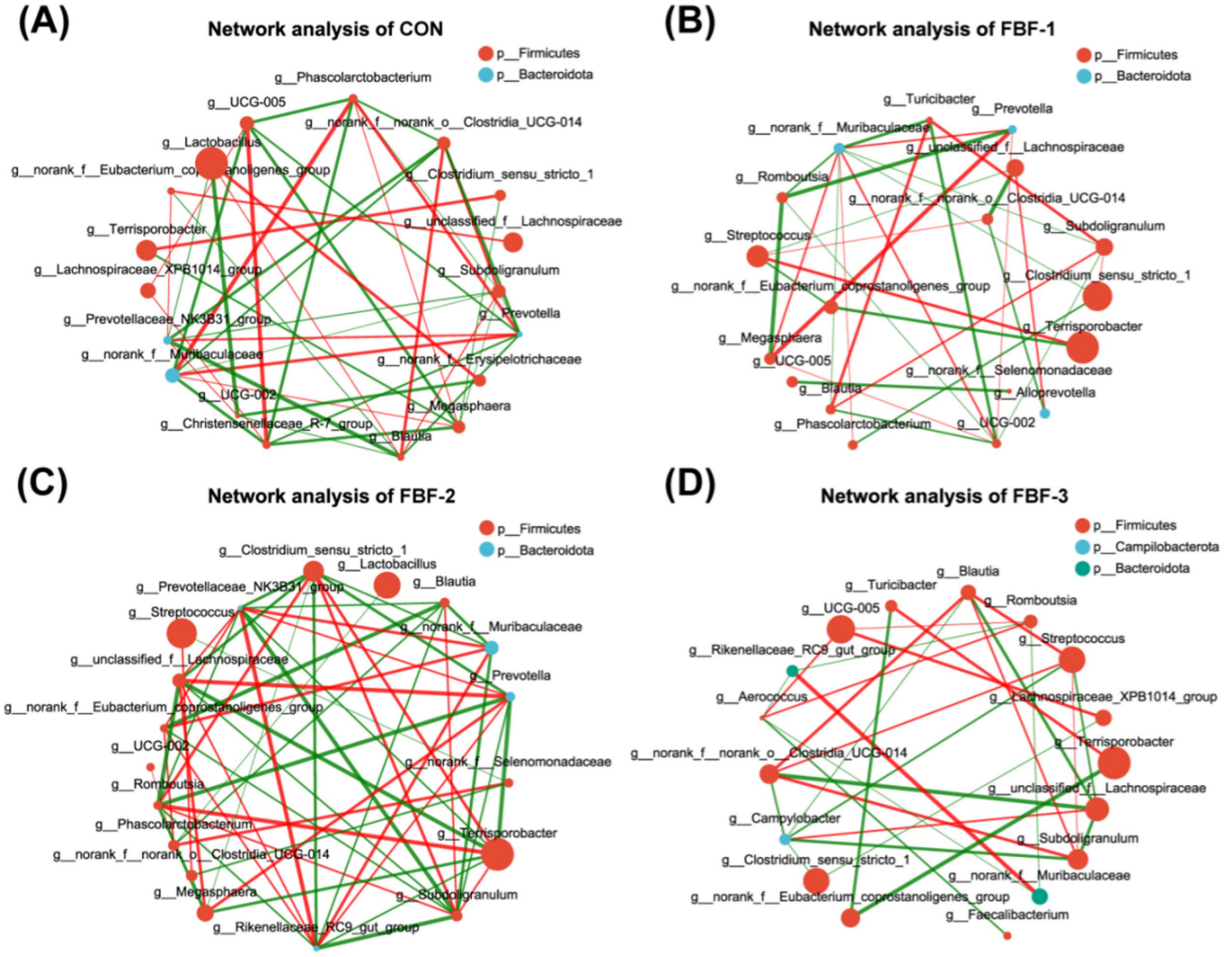
Network analysis of CON **(A)**. Network analysis of FBF-1 **(B)**. Network analysis of FBF-2 **(C)**. Network analysis of FBF-3 **(D)**. Single factor correlation network analysis of colon microbes. The size of the nodes indicates the abundance of species, and different colors indicate different species. Where red indicates a positive correlation and green indicates a negative correlation. The thickness of the line indicates the correlation coefficient. The thicker the line, the higher the correlation between species; the more lines, the closer the relationship between the species and other species.

Alpha diversity of the colonic microbiota in piglets, as influenced by fermented bamboo fiber, is presented in Figures 4A,B. Supplementation with fermented bamboo fiber did not significantly alter the richness or diversity of the colonic microbiota. Beta diversity, reflecting inter-sample differences in species diversity, is depicted in Figures 4E,F. PCoA and PCA analyses reveal a distinct separation between the microbiota of the different FBF groups and the control group, with significant differences observed (*p* < 0.05). Figures 4C,D illustrates the relative abundance of the dominant phyla and genera across the various groups. At the phylum level, *Firmicutes*, *Bacteroidota*, and *Actinobacteriota* were predominant in all groups. Notably, the FBF-3 group exhibited a significant increase in the proportion of *Verrucomicrobiota* (Figure 4J). Differential taxonomic analysis identified several genera with altered abundance, including *Clostridium sensustricto1*, *ChristensenellaceaeR-7 group*, *Turicibacter*, *Ruminococcus*, *Family XIII AD3011 group*, *Olsen-ella*, *Clostridium sensustricto6*, *Negativibacillus*, *Intestinibacter*, *Eubacterium nodatum group*, *norank_f__norank_o__Clostridia_vadinBB60 group*, *unclassified_f__Anaerovoracaceae*, *Candidatus Soleaferrea*, *Escherichia-Shigella*, and *Mogibacteri-um* (Figure 4G). Further analysis using LEfSe identified 22 distinct taxa between the control and experimental groups at an LDA score ≥ 3, encompassing 1 phylum, 1 class, 3 orders, 7 families, and 10 genera (Figure 4I). In the control group, enriched taxa included *g__norank_f__p-251-o5*, *f__p-251-o5*, and *g__Lachnospira* (Figure 4H). The FBF-1 group was enriched in butyrate-producing bacteria such as *o__Clostridiales*, *f__Clostridiaceae*, *g__Clostridium_sensustricto1*, and *g__Faecalibacterium* (Figure 4H). The FBF-2 group showed enrichment in butyrate-producing bacteria like *g__Clostridium_sensustricto6* and *g__Olsenella* (Figure 4H). The FBF-3 group was enriched with taxa such as *o__Oscillospirales*, *g__Turicibacter*, *f__Anaerovoracaceae*, *g__Rumi-nococcus*, *g__Family_XIII_AD3011 group*, *f__Bacteroidales_RF16 group*, *g__norank_f__Bacteroidales_RF16_group*, *p__Verrucomicrobiota*, *g__Eubacterium_ nodatum_group*, *c__Verrucomicrobiae*, *f__Akkermansiaceae*, *g__Akkermansia*, and *o__Verrucomicrobiales* (Figure 4H). These findings indicate that dietary supplementation with fermented bamboo fiber modulates the gut microbiota composition in weaned piglets. To further investigate the relationships among microbial species, a univariate correlation network analysis was performed on the 20 most abundant bacterial genera (Figure 5). The number of nodes with a degree ≥8 in the control group, FBF-1 group, and FBF-3 group were 2, 1, and 0, respectively (Figures 5A,B,D). In contrast, the FBF-2 group exhibited 7 nodes with a degree ≥8 (Figure 5C), and the positive/negative ratio of connections was higher in the FBF-2 group compared to the control group.

### 3.5 Effects of fermented bamboo fiber on serum factors related to excretion regulation and fecal pollutants

**Figure 6:**
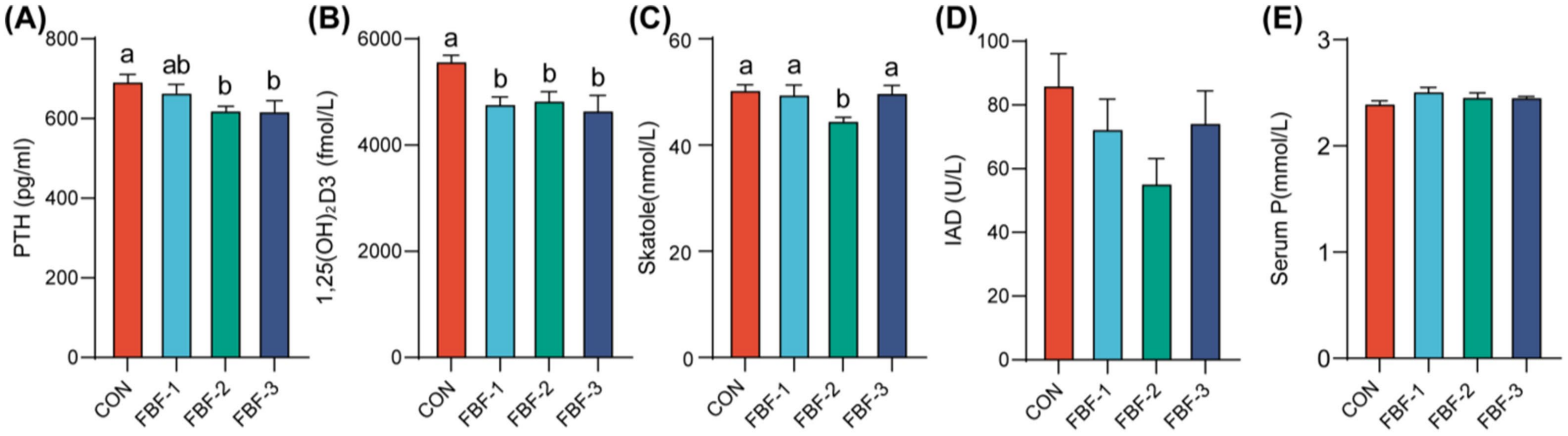
Serum indicators affecting the level of fecal contaminants in weaned piglets. **(A)** Parathyroid Hormone content; **(B)** 1,25(OH)_2_D_3_ content; **(C)** Skatole content; **(D)** Indoleacetic acid Decarboxylase content; **(E)** Serum Inorganic Phosphorus content. Different letters mean significant differences (*p* < 0.05).

**Figure 7:**
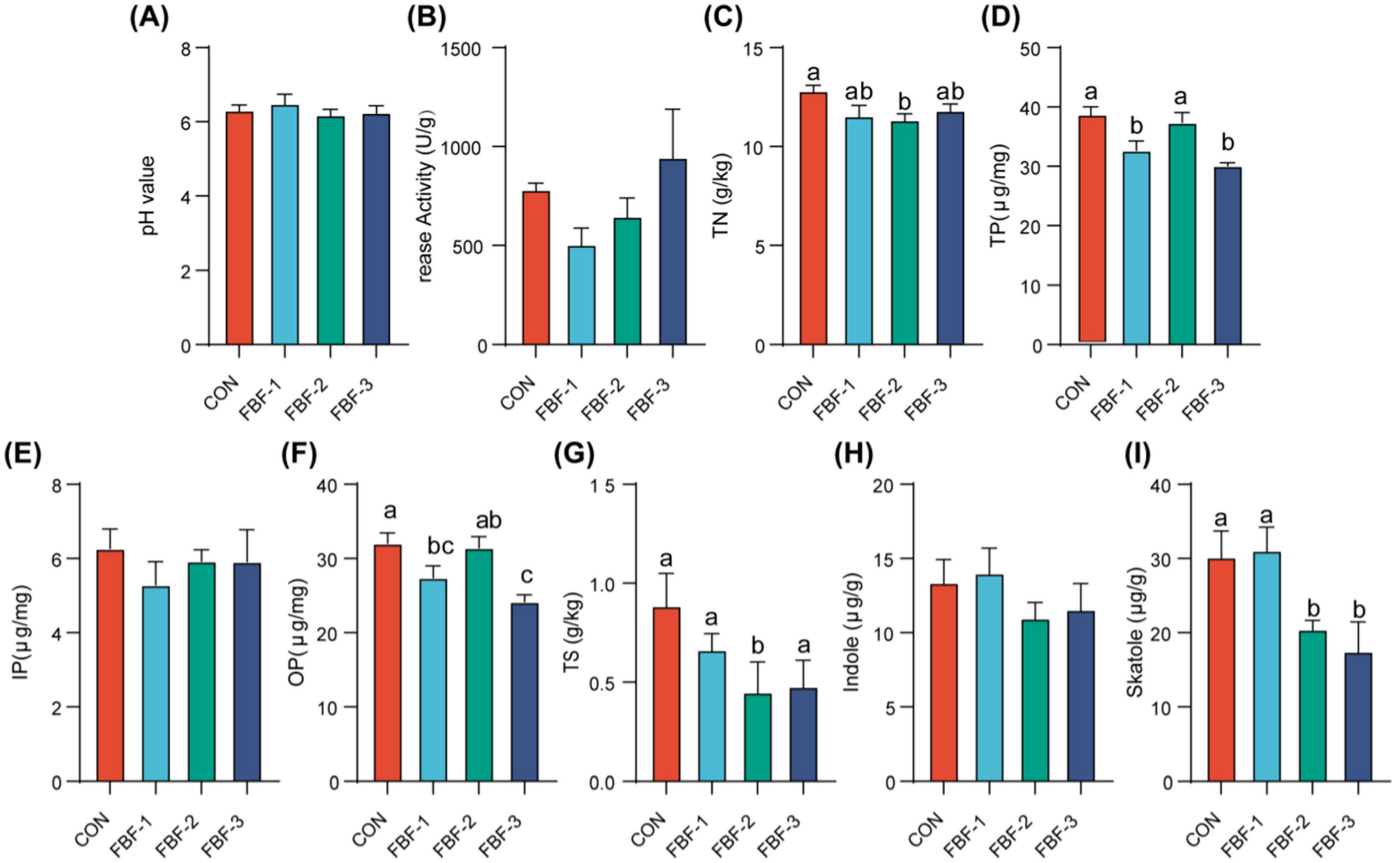
Effects of FBF supplementation on fecal excretion indicators of weaned piglets. **(A)** pH value; **(B)** Urease activity; **(C)** Total Nitrogen; **(D)** Total Phosphorus; **(E)** Inorganic Phosphorus; **(F)** Organophosphorus; **(G)** Total Sulfur; **(H)** Indole; **(I)** Skatole. Different letters mean significant differences (*p* < 0.05).

The results presented here suggest that dietary supplementation with fermented bamboo fiber modifies the colonic microbiota composition in piglets. We propose that this modification could lead to a reduction in pollutant excretion through feces. To investigate this hypothesis, we assessed serum factors involved in fecal regulation and analyzed fecal components in the piglets. As depicted in Figures 6A–E, with the exception of an increase in serum inorganic phosphorus levels in the experimental groups, there was a reduction in the levels of parathyroid hormone, 1,25-dihydroxyvitamin D3, skatole, and indoleacetic acid decarboxylase. Notably, 1,25-dihydroxyvitamin D3 levels were significantly decreased across all experimental groups. In addition, parathyroid hormone levels were significantly reduced in the FBF-2 and FBF-3 groups. The FBF-2 group, in particular, exhibited a significant reduction in skatole levels and showed the greatest decrease in indoleacetic acid decarboxylase among the experimental groups. Figures 7A–I details the impact of fermented bamboo fiber on fecal components. The results indicate that fermented bamboo fiber did not significantly affect fecal pH. While fecal urease activity was reduced in the FBF-1 and FBF-2 groups, these changes were not statistically significant. However, the FBF-2 group significantly reduced the total nitrogen and total sulfur content in feces. The FBF-1 and FBF-3 groups demonstrated a significant decrease in the total phosphorus and organic phosphorus content of feces. Additionally, skatole levels were significantly reduced in the feces of the FBF-2 and FBF-3 groups.

## 4 Discussion

This study investigated the effects of dietary supplementation with FBF on growth performance, intestinal barrier integrity, gut microbiota composition, and fecal pollutant levels in weaned piglets (Duroc × Landrace × Yorkshire). Our findings revealed that FBF supplementation significantly enhanced growth performance and intestinal barrier function, modulated the composition of gut microbiota, and reduced levels of fecal pollutants. These effects suggest potential long-term environmental benefits that warrant further investigation. Notably, a supplementation level of 1.5% was found to produce the most significant effects.

Previous studies have demonstrated that incorporating appropriate levels of dietary fiber into animal feed can enhance production performance and support intestinal health (38, 39). The effects of dietary fiber depend on its source, physicochemical properties, and fermentation characteristics (29, 40–42). In this study, supplementation with FBF improved the growth performance of weaned piglets and alleviated weaning stress. Although both the FBF-1 and FBF-2 groups exhibited increased average daily gain (ADG) compared to the control group, the differences were not statistically significant. However, the FBF-2 group showed a significant reduction in the feed-to-gain ratio (F/G). In contrast, the FBF-3 group experienced a significant decrease in ADG and an increase in F/G, suggesting that excessive supplementation can be detrimental. Considering the sensitivity of the weaned piglets’ intestines, this may lead to diarrhea, reduced feed intake, and damage to the intestinal barrier. These findings suggest that 1.5% FBF may be an optimal level to target. To test this hypothesis, we varied the levels of FBF and investigated their effects on the intestinal barrier function of weaned piglets.

In commercial production, many piglets experience weaning stress due to sudden separation from the sow, changes in diet and living environment, and other challenges. This stress negatively affects the morphology and function of the small intestine, disrupts digestion and absorption, and destroys intestinal barrier function, ultimately leading to reduced feed intake, increased diarrhea rate, and growth retardation (43, 44). Desai et al. (45) and Schroeder et al. (46) demonstrated that dietary fiber could enhance gut health by strengthening the intestinal barrier. Conversely, Shi et al. (47) found insufficient dietary fiber intake was associated with compromised intestinal barrier integrity. This disruption is usually marked by increased serum levels of D-lactate (D-LA), diamine oxidase (DAO), and endotoxin (ET) (48, 49). Our study demonstrated that FBF supplementation positively affected small intestinal morphology, with a 1.5% concentration yielding the most pronounced improvement. Furthermore, varying levels of FBF supplementation resulted in significant elevations in serum intestinal trefoil factor (ITF) levels, with notable reductions in intestinal fatty acid-binding protein (iFABP) levels observed in the FBF-2 and FBF-3 groups. The ITF is a protective peptide secreted by goblet cells and is detectable in both serum and the intestinal lumen. It plays a crucial role in maintaining mucosal permeability, with elevated levels of ITF indicating a strengthened intestinal barrier (50). In contrast, iFABP is secreted by damaged epithelial cells and serves as a marker of epithelial integrity. Increased levels of iFABP represent compromised epithelial integrity (51). Additionally, AB-PAS staining in this study revealed that the experimental groups had higher mucin content in the jejunum and ileum than the control group, with the FBF-2 and FBF-3 groups also exhibiting increased colonic mucin content. The FBF-2 group, in particular, showed significantly higher mucin levels across the jejunum, ileum, and colon compared to controls. Mucins are secreted by goblet cells and are critical for safeguarding epithelial cells from pathogenic invasion and for maintaining the integrity of the intestinal barrier (52). Dietary fibers are known to enhance this mucosal barrier by promoting mucus secretion, which provides a physical defense (45). The findings reported in this study are consistent with earlier reports that dietary fiber enhances goblet cell proliferation and mucin secretion (53). Overall, our results suggest that FBF supplementation in the diet markedly improves intestinal barrier function and gut health in weaned piglets, with a 1.5% concentration showing the most substantial benefits among the tested groups.

The gut microbiota is intricately connected to host health, playing a pivotal role in maintaining metabolic homeostasis as well as supporting a wide range of physiological, neurological, and immune functions (54, 55). Culture-independent techniques have revealed that the gastrointestinal tract (GIT) contains a dynamic microbial population with unique organisms residing in different sections (56, 57), and the most diverse group of microbes inhabit the colon in pigs (58). Moreover, dietary fiber is increasingly recognized for its role as a nutrient that interacts with the microbiota, influencing host health and immune responses (59).Dietary fiber has been implicated in modulating the intestinal microbiota and its metabolites, which may play a critical role in sustaining intestinal microecological balance and safeguarding gut health (60). Specific gut bacterial species (*Prevotella, Xylanibacter, Bacteroides thetaiotaoicum*) ferment indigestible fibers into short-chain fatty acids (SCFAs) such as acetate, propionate, and butyrate, thereby influencing host energy metabolism, immune regulation, and the integrity of the mucosal barrier (30, 61, 62). *Faecalibacterium prausnitzii*, a consumer of acetate and a butyrate producer, reduces the effect of acetate on mucus and prevents overproduction of mucus, thus maintaining an appropriate structure and composition of the gut epithelium. Evidence further suggests that butyrate, a microbial-derived metabolite, enhances the expression of tight junction proteins and promotes the differentiation of regulatory T cells (Tregs) within the colonic mucosa, contributing to gut homeostasis (30). Moreover, dietary fiber has been shown to alleviate intestinal inflammation caused by high-carbohydrate, low-fiber Western diets in murine models, in part by restoring the compromised intestinal mucus layer (63).The protective effects of fiber and resistant starch in experimental colitis are believed to rely on the production of SCFAs by the gut microbiota (29, 60). In light of this, the current study investigated the effects of FBF on gut health by analyzing the microbial composition of the colon in piglets. Principal Coordinate Analysis (PCoA) and Principal Component Analysis (PCA) revealed distinct clustering and significant differences in the gut microbiota between the FBF-treated groups and the control group. Across all groups, the predominant phyla were *Firmicutes, Bacteroidota,* and *Actinobacteriota*, consistent with previous findings regarding dominant gut microbiota in pigs (39, 64, 65). Comtet-Marre et al. (66) and Söllinger et al. (67) found that *Firmicutes* and *Bacteroidetes* in the gastrointestinal tract (GIT) are instrumental in the digestion of cellulose and hemicellulose. Additionally, research has shown that *Verrucomicrobia*, a mucus-degrading bacterium (68), tends to increase in abundance in patients with antibiotic-associated gut dysbiosis (69). The FBF-3 group exhibited a significant increase in the relative abundance of *Verrucomicrobia* compared to other groups, suggesting a potential weakening of the mucus barrier. In contrast, the 1% FBF and 1.5% FBF groups showed a significantly higher proportion of *Terrisporobacter* compared to the control (CON) and 2% FBF groups. *Terrisporobacter* is commonly found in suckling and weaning pigs and can produce short-chain fatty acids (SCFAs) from proteins. Its abundance in the GIT has been shown to be positively correlated with muscle weight gain in adult pigs (70). Moreover, FBF supplementation markedly increased the abundance of *Clostridium_sensu_stricto_1* in the intestines of piglets, a bacterium associated with the prevention of pathogenic bacterial colonization (71, 72). Higher levels of *Clostridium_sensu_stricto_1* are typically found in healthy piglets, while a decrease in its abundance has been linked to diarrhea in piglets (73). Additionally, FBF supplementation significantly increased the levels of *Clostridium_sensu_stricto_6*, *Christensenellaceae_R-7_group*, *Ruminococcus*, and *Olsenella*. The *Christensenellaceae_R-7_group* is implicated in immune regulation within the gut (74), whereas *Ruminococcus* and *Olsenella* are capable of fermenting cellulose to produce acetate and butyrate (75, 76). Interestingly, the 1.0 and 1.5% FBF groups showed a significant decrease in the abundance of *Family_XIII_AD3011_group*, while the 2.0% FBF group showed a significant increase. In light of previous research that a higher abundance of *Family_XIII_AD3011_group* may induce inflammation (77), these results indicate that excessive FBF supplementation could potentially impair immune function in weaned piglets.

LEfSe analysis revealed that the FBF groups were enriched with the dominant taxa *o__Clostridiales* and *o__Oscillospirales*. *o__Clostridiales* has the ability to utilize cellulases (78), hydrolyze cellulose, and inhibit pathogenic bacteria (79), whereas *o__Oscillospirales* is known to produce butyrate (80) and has been positively correlated with growth performance parameters (81). Additionally, 16S rRNA analysis demonstrated that FBF increased the abundance of SCFA-producing bacteria in the intestine. SCFAs, generated through bacterial fermentation of cellulose, enhance probiotic colonization and contribute to improved immune function and gut environment (82–85). Collectively, these results suggest that FBF supplementation alters the gut microbial composition of weaned piglets and exerts beneficial effects on gut health.

The intestinal microbiota serves as a key player in the digestion and absorption of nutrients in pigs. Alterations in the gut microbiota due to dietary supplementation can influence odor emissions from manure (86, 87). The main components of the emissions are Ammonia (NH_3_) and hydrogen sulfide (H_2_S) (88). H_2_S is a potent neurotoxin that can paralyze the olfactory nerves and cause ophthalmitis and respiratory tract inflammation in both humans and livestock (89). In contrast, NH_3_ is noxious and can irritate the mucosa of animals and cause inflammation (90). These manure emissions can have a detrimental effect on the environment. On top of that, livestock and poultry manure is often characterized by its high phosphorus content and widespread use as fertilizer, which increases phosphorus fluxes within ecosystems and exacerbates nutrient pollution in natural environments (91). Therefore, mitigating the environmental impact of manure necessitates a reduction in the levels of nitrogen (N), phosphorus (P), and sulfur (S) in the waste. In this study, dietary supplementation with FBF reduced total nitrogen (TN), total phosphorus (TP), and total sulfur (TS) in the feces compared to the control group, with the most notable decreases observed at 1.5% FBF for TN and TS, and at 1 and 2% FBF for TP. Additionally, previous research has highlighted the interaction between parathyroid hormone (PTH) and 1,25-dihydroxyvitamin D_3_ [1,25(OH)_2_D_3_] in maintaining phosphorus homeostasis and intestinal phosphorus absorption (92). To elucidate the mechanisms underlying the observed reduction in fecal phosphorus with FBF supplementation, we analyzed serum levels of phosphorus (P), PTH, and 1,25(OH)_2_D_3_. The results showed that FBF supplementation slightly increased serum phosphorus levels and decreased PTH levels, particularly in the FBF-2 and FBF-3 groups, along with a marked reduction in 1,25(OH)_2_D_3_ levels across all FBF groups. These findings contrast sharply with those of Shah et al. (28), who reported that in a high-fiber (HF) diet group, serum phosphorus levels were marginally lower, and PTH levels were slightly higher compared to a medium-fiber diet group, although these differences were not statistically significant. Based on our findings, we hypothesized that the FBF-induced reduction in fecal phosphorus excretion may be attributed to enhanced phosphorus reabsorption. This effect is likely mediated by decreased concentrations of 1,25(OH)_2_D_3_ and PTH, which together contribute to decreased phosphorus excretion (93). Additionally, our study showed that FBF supplementation in the diet significantly decreased skatole levels in feces. Indole and skatole are the two primary final products of intestinal bacteria. Among them, skatole is more easily noticeable due to its prevalence in animal feces, wastewater, and sewage sludges, where its concentrations can reach up to 72.2 mg/kg (94). Moreover, skatole is detectable at extremely low concentrations, with a threshold odor level of just 0.00056 ppm (95). Li et al. (96) demonstrated that highly fermentable dietary fibers, such as those found in chicory root, can lower skatole production in boars. Su et al. (97) also found that fermented herbal residues can significantly reduce indole levels in the colonic contents of weaned piglets. Our results in this study are consistent with these previous findings. Additionally, Liu et al. (95) and Fu et al. (98) described the biochemical pathway for skatole production, indicating that indole-3-acetic acid can be converted into skatole through the action of IAD. Building on this knowledge, we examined the effects of FBF on serum IAD concentrations and found that FBF supplementation reduced serum IAD levels, although this decrease was not statistically significant. Notably, the serum skatole levels were significantly reduced in the FBF-2 group, mirroring the changes in IAD levels across all groups, with the most marked reduction occurring in this group. These preliminary findings suggest that FBF may effectively reduce the excretion of nitrogen (N), phosphorus (P), sulfur (S), skatole, and indole in piglet feces, highlighting its potential to diminish fecal odor and mitigate environmental pollution.

## 5 Conclusion

This study demonstrated that FBF supplementation in weaned piglet diets effectively mitigated weaning stress by enhancing intestinal morphology, barrier function, and microbiota composition. Future research should focus on the long-term effects of FBF and its broader application in livestock production. These modifications facilitated beneficial bacterial colonization and immunological development. Furthermore, FBF reduced fecal pollutants and manure odor emissions, contributing to environmental sustainability. While preliminary, this work provides valuable insights into strategies for alleviating weaning stress, boosting piglet immunity, and minimizing the environmental footprint of livestock production. Future research can build upon these findings to further elucidate the underlying mechanisms by which FBF affects swine production.

## Data Availability

The original contributions presented in the study are included in the article/supplementary material, further inquiries can be directed to the corresponding author.

## Footnotes

^1^ https://www.majorbio.com
